# Case report: Gemcitabine intravesical hyperthermic infusion combined with tislelizumab in muscle invasive bladder urothelium carcinoma

**DOI:** 10.3389/fonc.2022.1062655

**Published:** 2022-12-23

**Authors:** Zheng Du, Huaqi Yin, Shiming Zhao, Yongkang Ma, Zhenghui Sun, Bingqi Dong, Mingkai Zhu, Chaoshuai Zhu, Jiangshan Peng, Tiejun Yang

**Affiliations:** Department of Urology, The Affiliated Cancer Hospital of Zhengzhou University, Henan Cancer Hospital, Zhengzhou, China

**Keywords:** muscle invasive bladder urothelium carcinoma, chemotherapy, bladder hyperthermia, gemcitabine, tislelizumab, tumor immunotherapy

## Abstract

**Background:**

Muscle invasive bladder urothelium carcinoma is a common urinary tract tumor. With the deepening of research, more and more treatment methods are applied in clinical practice, extending the life of patients. Among them, the clinical application of chemotherapeutic intravesical hyperthermia and tumor immunotherapy provides new ideas for our treatment.

**Case report:**

An 81-year-old female patient was diagnosed with stage T2N0M0 bladder cancer in our hospital. Because the patient and her family were keen to preserve her bladder, they declined surgery and opted for combined chemotherapy. After informed consent from the patient and her family, she received cisplatin combined with gemcitabine intravesical hyperthermic infusion. But the side effects of cisplatin made her intolerable to chemotherapy. With their informed consent we changed her to intravenous tislelizumab in combination with gemcitabine intravesical hyperthermic infusion to continue her treatment. During the subsequent follow-up visits, we found a surprising effect of the treatment.

**Conclusion:**

Gemcitabine intravesical hyperthermia therapy combined with intravenous tislelizumab in the treatment of muscle invasive bladder urothelium carcinoma may provide a new possible therapeutic strategy of some patients who are inoperable or refuse surgery.

## 1 Introduction

With the further research, the treatment of muscle-invasive bladder cancer has brought hope to more and more patients. The non-operative treatment of muscle-invasive bladder cancer includes chemotherapy, hyperthermia and immunotherapy (1). The experimental results show that hyperthermia can synergize with chemotherapy and radiotherapy to a certain extent and improve its efficacy (2, 3). Clinical studies have also shown that this is an effective way to prolong the survival time of patients and reduce the incidence of total cystectomy (4). Intravesical chemotherapy combined with hyperthermia is expected to become standard of care in patients who refuse radical cystectomy (5). Rise of immunotherapy brings new treatment options and hope to cancer patients (6). However, the dilemma was that the objective response rate of ICIs (immune checkpoint inhibitors, ICIs) alone is low in various carcinomas (7). Therefore, how to improve the objective response rate of immunotherapy is an urgent problem to be solved in current immunotherapy. At present, chemotherapy drugs combined with intravesical hyperthermia and immunotherapy are not widely used in clinical practice. Next, we report a case of T2N0M0 bladder urothelial carcinoma treated with gemcitabine bladder hyperthermia infusion combined with immunotherapy drug tislelizumab.

## 2 Case description

**Figure 1:**
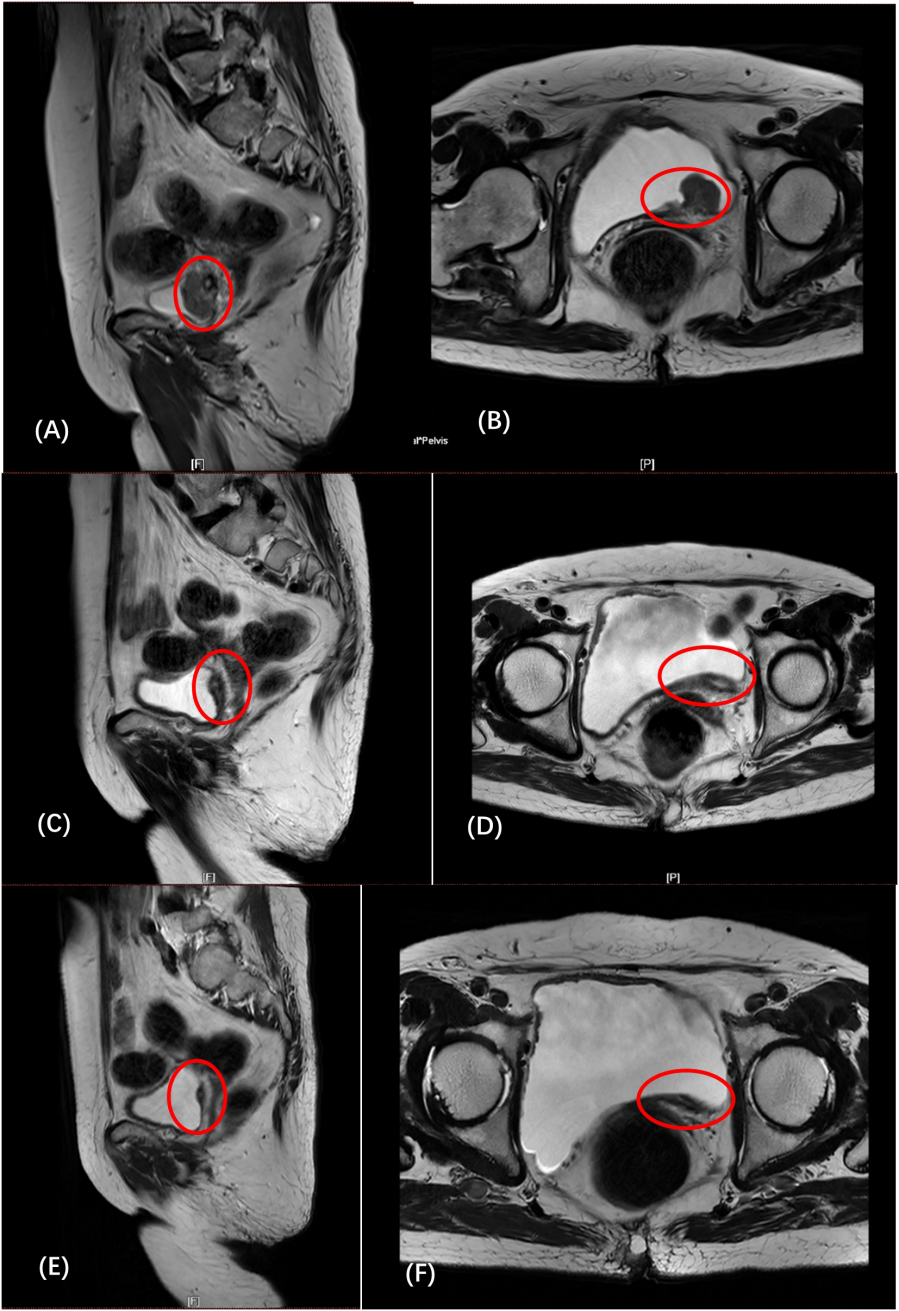
**(A, B)** (February 6): the left posterior wall of the bladder was unevenly thickened, and abnormal signal nodules were seen. **(C, D)** (April 3): compared with before, the tumor has shrunk significantly, and the lymph nodes are similar to before. **(E, F)** (May 26): The thickness of the left posterior wall of the bladder was reduced, and the lymph nodes remained unchanged.

**Figure 2:**
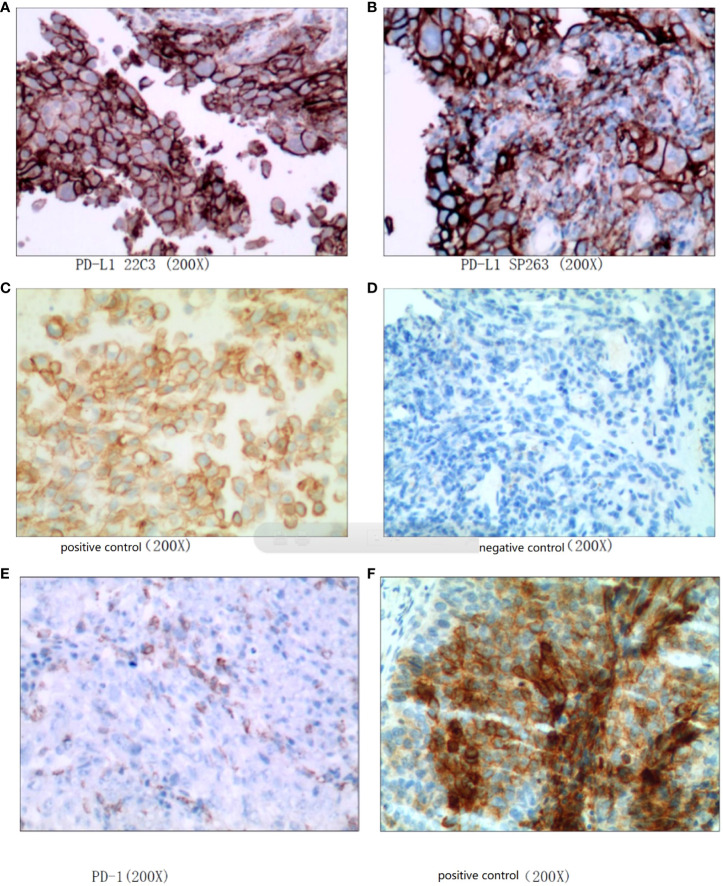
High-grade urothelial carcinoma. **(A–D)**: PD-L1 (CPS: 70), **(E, F):** PD-1 (positive tumor cell number 5%).

An 81-year-old woman was initially referred to our ward due to mild urinary frequency, urgent urination and painful urination that had lasted for 1 month. One month prior to referral, the patient had new-onset urinary frequency, urgent urination and painful urination without obvious inducement. During this month, the patient was treated with antibiotics at the local hospital, but the response was not satisfactory. After a CT examination at the local hospital, a bladder mass was found and then referred to our ward. The patient has suffered from hypertension and diabetes for 30 years, and her blood pressure is currently controlled within the normal range after oral medication. Laboratory investigations included the following: leukocytes: 4.31 × 10^9^/L, hemoglobin: 128g/L; blood sugar: 8.45 mmol/L (reference range: 3.9-6.1umol/L); triglycerides: 3.55mmol/L (reference range: 0-1.7mmol/L); platelet :113×10^9^/L (reference range: 125-350×10^9^/L). Routine urine examination: urine occult blood: +-cell/UL; White blood cell: +-cell/UL; White blood cell:40/ul (reference range: 0-28/ul). Ultrasound examination showed a 34×21 mm solid mass in the left anterior wall of the bladder. MRI report (**Figures 1A, B**): there is a 24×16×29 mm nodule in the left posterior wall of the bladder that can be obviously unevenly enhanced, no enlarged lymph nodes. Cystoscopy and biopsy confirmed high-grade bladder urothelial carcinoma. Pathology report (**Figure 2**): High-grade urothelial carcinoma, HER- 2 protein: (2+), PD-1 (positive tumor cell number 5%), PD-L1 (Combined Positive Score (CPS): 70). Preliminary diagnosis: 1. Bladder urothelial carcinoma T2N0M0; 2. High risk of hypertension grade 1; 3. Type II diabetes. Despite our patient communication, patients and their families still want to preserve the bladder and refuse surgery.

After fully communicating with the patient and his family and signing the informed consent, use the gemcitabine + cisplatin regimen for treatment. On February 14, 2022, the patient received intravenous cisplatin for the first time, and on February 15, she received gemcitabine intravesical hyperthermia under local anesthesia. Subsequently, the patient developed intolerance symptoms such as nausea, vomiting, and fatigue. We considered the patient to be intolerant to platinum-based chemotherapy. After the patient and his family agreed and signed the informed consent, the treatment plan was changed to gemcitabine intravesical hyperthermic infusion combined with intravenous tislelizumab immunotherapy. On March 4, 2022, the patient received gemcitabine intravesical hyperthermia under local anesthesia and received intravenous tislelizumab for the first time. The patient subsequently received gemcitabine intravesical hyperthermic infusion combined with intravenous tislelizumab every three weeks. The patient received a total of 5 complete treatments of gemcitabine bladder hyperthermia combined with intravenous tislelizumab. On April 3, the patient was reexamined MRI (**Figures 1C, D**), and the report: compared with before, the tumor has shrunk significantly, and the lymph nodes are similar to before. ECT report: no obvious abnormal bone metabolism was found in the whole body. On May 26, she reviewed the MRI (**Figures 1E, F**): The thickness of the left posterior wall of the bladder was reduced, and the lymph nodes remained unchanged. The patient developed symptoms of bladder infection after the end of treatment in June, and subsequently received only intravenous tislelizumab intravenous infusion, but failed to receive bladder hyperthermic infusion therapy.

## 3 Discussion and conclusion

In this case, we observed that gemcitabine intravesical hyperthermia combined with intravenous tislelizumab brought a good antitumor effect to the patient, and the tumor responded completely. What is even more commendable is that after treatment, the idea of preserving the bladder for the patient is satisfied, while avoiding the trauma and psychological damage caused by the operation. This also fully demonstrates the feasibility of this combination therapy.

As a pyrimidine antimetabolite, gemcitabine is usually used in the systemic treatment of various malignant tumors (8). With the in-depth study of hyperthermia combined with chemotherapy, it can be confirmed that hyperthermia has an encouraging effect in the treatment of bladder cancer (9). Studies have shown that intravesical hyperthermic perfusion play a synergistic effect with a variety of chemotherapy drugs in the application of bladder cancer. Compared with ordinary infusion chemotherapy, intravesical hyperthermic infusion can reduce the recurrence rate, reduce the incidence of radical cystectomy, and has higher safety (4, 9, 10). Some scholars have reported that compared with passive diffusion, hyperthermia can increase the delivery concentration of the drug, shorten the delivery time and increase the concentration of the drug in the urothelium (11). Hyperthermia prevents cisplatin-induced DNA damage-induced slowing of replication forks, enhances the formation of double-strand breaks in replicating cells, significantly delays cisplatin-induced DNA damage repair, and blocks chemotherapy-induced polyADP-ribosylation (PARyla-tion) (12). Based on this, we selected a thermal perfusion equipment that adopts computer numerical control heating system, automatic safety guarantee system, non-interference temperature measurement system and active automatic cooling system. During patients’ treatment, the device utilizes water bath heating and efficient heat exchange to precisely control the temperature within 43 ± 0.1°C (4). Unfortunately, our patient did not tolerate cisplatin chemotherapy due to severe side effects. Even compared with other platinum-based drugs, cisplatin has fewer side effects than carboplatin in the treatment of bladder tumors (13). In some reports, we have collected some side effects of cisplatin, such as hepatotoxicity, cardiotoxicity, nephrotoxicity, and allergic reactions (14).

In subsequent treatments, tislelizumab replaced cisplatin. Tislelizumab was approved in China in April 2020 for patients with locally advanced or metastatic urothelial cancers who have previously received platinum-based treatment. In recent years, immunotherapy has shown broad application prospects in the field of tumor treatment and has become a new hope for conquering tumors. Tislelizumab was evaluated in a single-arm phase 2 study (NCT04004221/CTR20170071) to determine whether it is safe and effective in patients with PD-L1-positive urothelial cancer. 104 patients were included in the efficacy assessment for antitumor activity. Confirmed objective responses were observed in 25 patients (overall objective response rate, 24%, 95% CI, 16, 33) (15). The abnormal expression of immune checkpoints in the tumor microenvironment makes the immune response unbalanced and the anti-tumor immune response is inhibited. Tislelizumab is a ICIs that can block the binding of the immune checkpoint PD-1 to its ligands. It can improve anti-cancer immune activity and has good application prospects in the field of tumor therapy (16). Although ICIs can bring certain survival benefits to some patients and maintain a sustained immune response for a long time. The results of several studies have shown that the overall objective response rate (ORR) of ICIs alone is low, only 15%-31% (17, 18). In related reports, scholars have proposed that hyperthermia leads to the release of heat shock proteins, especially HSP70, which stimulate adaptive T cell responses and induce innate and adaptive immunity (19). Hyperthermia-induced HSP70 can initiate anti-tumor immune responses by inducing tumor cells to secrete cytokines. After knocking out the HSP70 gene, mouse tumor growth was accelerated, and a lack of immune cell infiltration in tumor tissue could be observed (20). In addition, Kolosnjaj-Tabi et al. showed that hyperthermia can reduce tumor stiffness and soften its extracellular matrix, thereby promoting drug penetration and immune cell infiltration by lowering the physical barrier effect (21). Some scholars have reported that gemcitabine can activate immunogenicity of tumor cells and increase the efficacy of immunotherapy (22). Tislelizumab is an immunotherapy drug. We hope to enhance the effect of immunotherapy with hyperthermia. Fortunately, the therapeutic effect of this line is fully reflected in this patient

The quality of life and life burden of bladder cancer patients after surgery may lead to psychological distress. In one study, scholars found that patients had significant psychological distress that persisted for one month after receiving RC. In one study, scholars found that a considerable proportion of patients had psychological distress in the perioperative period of RC (23). The woman we treated avoided RC, and a positive mindset may have helped her recover as well.

Finally, some side effects in this treatment plan are also worth thinking about. The patient developed a urinary tract infection, which interfered with perfusion therapy. In the future, we should take more preventive measures to avoid urinary tract infections, including the use of silver ion or antibiotic-coated catheters, the use of more lubricants to reduce urethral friction and more delicate manipulations, etc. (24). Moreover, at the public level, the unacceptability of hyperthermia is also a current dilemma. One of the reasons is that some small organizations make these hyperthermia devices, and they lack mass media publicity and funding to support clinical trials (25). We should invest more research in the future to benefit more patients.

## Data availability statement

The original contributions presented in the study are included in the article/supplementary material. Further inquiries can be directed to the corresponding author.

## Ethics statement

Written informed consent was obtained from the individual(s) for the publication of any potentially identifiable images or data included in this article.

## Author contributions

TY and HY designed the study. HY and ZD were major contributors in writing the manuscript. SZ performed cystoscopy for the patient. YM and ZS treated the patient. BD, MZ, CZ was responsible for follow-up and recording. JP collected references. All authors contributed to the article and approved the submitted version.
